# MNT and Emerging Concepts of MNT-MYC Antagonism

**DOI:** 10.3390/genes8020083

**Published:** 2017-02-20

**Authors:** Guang Yang, Peter J. Hurlin

**Affiliations:** 1 Shriners Hospitals for Children Research Center, Portland, OR 97239, USA; guy@shcc.org; 2 Department of Cell, Developmental and Cancer Biology, Oregon Health & Science University, Portland, OR 97239, USA; 3 Knight Cancer Institute, Oregon Health & Science University, Portland, OR 97239, USA

**Keywords:** MNT, MYC, MAX, MLX, MXD, MLXIP, MYC antagonism

## Abstract

MYC family proteins play fundamental roles in stem and progenitor cell homeostasis, morphogenesis and cancer. As expected for proteins that profoundly affect the fate of cells, the activities of MYC are regulated at a multitude of levels. One mechanism with the potential to broadly affect the activities of MYC is transcriptional antagonism by a group of MYC-related transcriptional repressors. From this group, the protein MNT has emerged as having perhaps the most far-reaching impact on MYC activities. In this review, we discuss the current understanding of MNT, its regulation and how, as a MYC antagonist, it functions both as a tumor suppressor and facilitator of MYC-driven proliferation and oncogenesis.

## 1. Introduction

The importance of MYC in cancer was first recognized when it was found that the oncogenic properties of several acutely transforming chicken retrovirus strains were conferred by the virally transduced *c-MYC* (*MYC*) gene [1,2]. Genome alterations in *MYC* (MYC, MYCN and MYCL, collectively referred to as MYC) family genes, particularly gene amplifications, are now recognized as common features of a wide variety of cancers. These events, which lead to deregulation and/or abnormally high levels of MYC in cells, appear to be causal since their experimental recapitulation in mouse models leads to cancer, and these cancers are typically dependent on continued high-level or deregulated MYC expression [3]. In addition to anomalies at *MYC* loci, deregulated MYC expression can result from mutations, amplification and translocations of genes encoding various growth-factor receptors and signal transduction proteins that normally control MYC abundance and activity in cells. Such events can alter MYC transcription, translation and various post-translational modifications in MYC that control its stability and its function as a transcriptional factor. Together, the available evidence suggests that deregulated MYC expression is one of the most pervasive mechanisms underlying the development of cancer [4].

MYC interacts with the protein MAX through their shared basic-loop-helix-leucine zipper domains (BHLHZip) to bind DNA at the sequence CACGTG and related “Ebox” sequences and upregulate a large number of genes [5,6]. An increase in the collective output of this transcriptional program is thought to be responsible for much of the oncogenic activity of deregulated MYC. MAX also interacts with a group of transcriptional repressors which can antagonize the transcriptional activity of MYC-MAX complexes and may therefore function as tumor suppressors. This review focuses on our current understanding of one of these proteins, MNT, which may impinge not only on the activities of MYC, but also on those of other broadly acting transcription factors.

## 2. Transcriptional Antagonism between MYC and MNT

MNT (MAX’s Next Tango) was originally identified in a protein–protein interaction screen designed to detect proteins that interacted with MAX [7]. At about the same time, the *MNT* gene (previously referred to as *ROX*) was identified during chromosomal mapping studies looking for genes that might contribute to Miller-Dieker lissencephaly [8,9], a congenital malformation disorder characterized primarily by lissencephaly (smooth brain) [10]. While it remains possible that loss of *MNT* contributes to Miller-Dieker lissencephaly [11], its identification led to studies characterizing the MNT protein [9].

Like MYC, MNT was shown to heterodimerize with MAX through their related BHLHZip domains [7,9]. Both MYC-MAX and MNT-MAX heterodimers bind Ebox sequences, but whereas MYC-MAX typically increases transcription, MNT-MAX appears to repress transcription [7,9]. MNT and the related BHLHZip proteins MXD1–4 repress transcription through the recruitment of the corepressor SIN3 [7,9,12,13,14]. SIN3 (SIN3A and SIN3B) recruits a number of distinct proteins that appear to act together to repress transcription [15]. Included in this group are the histone deacetylases HDAC1 and HDAC2, which contribute to transcriptional repression by removing acetyl groups on histones and generating a more closed and inaccessible chromatin environment. In contrast, binding of MYC-MAX complexes to Ebox elements promotes transcription through MYC-dependent recruitment of one or more of an extensive list of potential coactivators that have been described as binding to MYC [16]. Generally, MYC coactivators act by modifying histones to generate a more open or relaxed chromatin environment and by associating with components of the basal machinery to stimulate polymerase activity [16]. Among the cofactors that MYC is known to recruit are histone acetyl transferases (HATs), which function in opposition to the HDAC activity associated with MNT and MXD family proteins. The predicted transcriptional antagonism between MYC-MAX and MNT-MAX or MXD-MAX has been described for a number of shared target genes [17,18,19,20,21,22].

Despite the identification of a number of genes that can be regulated by MYC-MNT antagonism, early genome-wide binding studies suggested that a substantial number of genes bound by MYC are also bound by MNT [23]. Moreover, comparison of gene expression signatures in cells overexpressing MYC and cells lacking MNT show some similarities but suggested limited overlap [23]. Similar observations were made in *Drosophila*, where dMNT and dMYC appear to co-regulate a subset of genes that are particularly enriched in rRNA synthesis and processing and are critical for *Drosphila* growth and viability [24,25,26]. More recent MNT ChIP-seq data compiled in The Encyclopedia of DNA Elements (ENCODE) project [27] (GEO:GSE91968) indicate that MNT binding occurs at proximal promoter locations of a larger number of genes than our earlier studies suggested, and that MNT binding overlaps with MYC binding at most, but not all MYC binding sites. These latter findings need further analyses, but the picture emerging is one where MNT-MAX and MYC-MAX are continuously competing for binding at hundreds or thousands of shared target genes, but that they each also have an extensive subset of unique, non-overlapping target genes.

Why might MYC and MNT have both shared and unique target genes? There are several potential reasons. First, the DNA binding basic regions of MYC and MNT are not identical and therefore are likely to have different affinities and preferences for DNA. Indeed, DNA binding assays indicate that MNT-MAX has some unique preferences and may not recognize or have reduced affinity for some Ebox sequences that MYC-MAX and MXD-MAX are known to recognize [9]. Further studies using ChIP-seq and related technologies will be useful in determining more precisely the in vivo DNA binding preferences of MNT-MAX complexes and how they may differ from those of MYC-MAX and MXD-MAX complexes.

**Figure 1:**
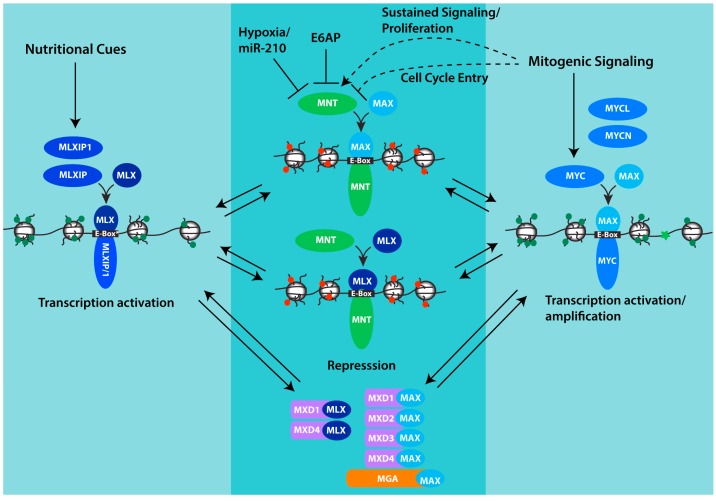
MNT and its place in the MAX-MLX network of interacting proteins. Through its interaction with both MAX and MLX, MNT is positioned to compete with both MLXIP/MLXIP1 and MYC family proteins for interaction with MLX and MAX respectively, and for binding to shared target genes. Whereas MYC and MLXIP complexes promote transcription, MNT complexes repress transcription. Chromatin is depicted to indicate histone modifications and architecture that acts to promote transcription (green marks, relaxed conformation, MYC-MAX and MXLIP/1-MLX) or repress transcription (red marks, closed conformation, MNT-MAX, MNT-MLX). MXD-MAX, MXD-MLX and MGA-MAX complexes also repress transcription and may function in concert with MNT complexes to antagonize transcription by MYC and MLXIP complexes and fine-tune the expression of shared target genes. For MNT, its abundance can be downregulated by hypoxia and specifically through the inhibitory mRNA binding by miR-210 which is strongly induced by hypoxia. MNT can also be downregulated by the ubiquitin ligase E6AP. The effect of mitogenic signaling on MNT appears variable, with evidence that MNT is significantly increased in settings of sustained proliferation, but that in the setting of growth-factor-induced cell cycle entry, phosphorylation of MNT by ERK acting downstream in the MAPK pathway transiently interferes with MNT binding to SIN3 corepressors and its ability to antagonize transcription by MYC. See text for additional details.

A second factor that may influence DNA binding specificity is the DNA binding partner of MNT. In addition to MAX, MNT can bind MAX-Like Factor X (MLX) to form complexes that bind Ebox sequences [28]. Like MAX, MLX is widely expressed [28,29] and while it has yet to be demonstrated, it seems likely that DNA binding preference and affinity by MNT-MAX and MNT-MLX will not be identical and therefore generate unique subsets of MNT target genes that may or may not overlap with MYC-MAX or MXD-MAX target genes. Despite the high degree of similarity between the four MXD family members, only two family members (MXD1 and MXD4) have been demonstrated to interact with both MAX and MLX [29]. The molecular mechanism responsible for this selectivity and its functional significance remains unclear. However, MLX also binds MLX interacting protein (MLXIP, formerly MONDOA) and MLX interacting protein 1 (MLXIP1, formerly CHREBP) to form complexes that activate transcription [30,31], and it is possible that repressive MNT-MLX and MXD-MLX complexes act to selectively antagonize MLXIP/MLXIP1-MLX-driven transcription and MNT-MAX and MXD-MAX complexes selectively act to antagonize MYC-MAX-driven transcription (Figure 1). MLXIP and MLXIP1 play important roles in nutrient sensing and regulating glycolysis and lipogenesis [32]. Interestingly, recent studies indicate that MYC and MLXIP co-regulate the expression of at least some shared target genes [33] and thus raise the possibility that MAX or MLX cofactor binding may have a limited effect on target gene selection. Studies to determine if and how target gene selection is determined by MNT’s binding partner and whether there is overlap in MNT-MLX and MLXIP-MLX binding sites and target gene regulation may help resolve questions concerning the significance of MNT binding to both MLX and MAX.

## 3. MNT as a Determinant in Non-Uniform Target Gene Responses to MYC

Genome-wide MYC binding studies combined with transcriptome analyses in cellular settings of controlled MYC expression indicate that increased MYC abundance corresponds to increased binding at its many binding sites (typically at gene promoter-proximal sites) and increased target gene expression [34,35]. MYC-MAX binding appears to preferentially occur at promoter regions where RNA polymerase and key components of the RNA polymerase complexes have already bound and transcription is active or poised to become active [36,37]. While MYC may act to increase productive transcription at many of its target genes, its effects on gene transcription are non-uniform [38,39]. Alternative modes of transcriptional regulation by MYC, including through interaction with MYC-interacting zinc finger protein (MIZ1/ZBTB17), together with differences in gene-specific target site binding affinity and dynamics by MYC-MAX complexes are at least two mechanisms that impart significant variation in transcriptional responses to MYC [40,41]. Another mechanism that likely modulates MYC-dependent transcription and causes variation in transcription at MYC target genes is competition between MNT-MAX and MYC-MAX. For example, differential binding affinity of MYC-MAX vs. MNT-MAX at shared target genes, or the lack of binding by MNT-MAX or MNT-MLX complexes at some but not all MYC-MAX targets, has the potential to generate considerable diversity in the profile of MYC-dependent gene expression as a function of MYC abundance. Additional layers of non-uniformity in MYC target gene responses to changes in MYC levels is likely conferred through differential binding to MYC target genes by repressive MXD and the additional MAX-binding BHLHZIP protein MGA (MAX’s Giant Associated protein [42]), and by MLXIP/1 activating complexes (Figure 1). Further, differential changes in the abundance of MNT, MXD1–4, MGA and MLXIP/1 relative to MYC in response to external cues has the potential to cause changes in the expression of target genes that they share with MYC in ways far more complicated that the current models account for.

## 4. MNT as a Tumor Suppressor

In addition to being able to antagonize transcription by MYC, forced MNT expression was found to block MYC-dependent transformation of mouse embryo fibroblasts [7]. In contrast, knockdown or deletion of *MNT* partially phenocopied the transformed state of cells subjected to ectopic MYC expression [17,18]. Beyond cell culture models, both experimental mouse models and interrogation of the *MNT* locus in human tumors provide supporting evidence that MNT may be a bonafide tumor suppressor. While germline deletion of *MNT* is typically neonatal or embryonic lethal depending on the genetic background [11], conditional deletion of *MNT* in either breast epithelium or T cells led to tumor formation [17,23,43]. Ectopic expression of MYC in these settings also leads to cancer and comparing expression profiling of breast tumors generated by MYC overexpression to MNT loss indicated significant overlap and potentially similar underlying mechanisms leading to tumor formation [23].

Chromosome loss at 17p13.3, where *MNT* is located, is observed in a variety of cancers and this locus is thought to contain one or more tumor suppressors [44,45]. Recent evaluation of tumor types cataloged within The Cancer Genome Atlas (TCGA) tumor database confirm that heterozygous loss of *MNT* is a prevalent event in cancer (TCGA Cancer Atlas Project, MYC Subgroup, in preparation). Perhaps the best documented connection between *MNT* loss and cancer is with the variant form of cutaneous T cell lymphoma known as Sézary Syndrome (SS). In two studies examining copy number variation (CNV) in SS, heterozygous loss of *MNT* was found in 55% of (11/20) patient samples [46] and 66% (38/58) patient samples [47] respectively. When combined, these studies show that 49% (38/78) of patients’ tumors had *MYC* gain, and 63% (49/78) had heterozygous *MNT* loss. In aggregate, 81% of SS tumor samples showed either heterozygous loss of *MNT* or *MYC* gain [47].

While *MNT* loss is thought to be an informative and important event in SS tumors, it should be noted that loss of *TP53*, which is located at 17p13.1, was found in 15 of 20 cases of SS in the original study examining *MYC* and *MNT* CNV [46]. TP53 CNV was not examined in the second study [47]. In tumor samples lacking both *TP53* and *MNT*, it is not known whether *TP53* and *MNT* loss are distinct events or are physically connected. Thus, data from SS tumors do not rule out the possibility that *MNT* loss is a passenger event in these tumors. However, given the experimental evidence showing that conditional deletion of *MNT* in T cells is oncogenic [43], it seems reasonable to surmise that *MNT* aberrations in SS likely contribute to tumor formation. Moreover, loss of the *MNT* related gene *MXD1* (formerly *MXI1*) was found in 8/20 SS tumor specimens, suggesting that loss of *MYC* antagonism is a common contributing mechanism in SS tumorigenesis [46].

Assuming that loss of *MNT* and gain of *MYC* observed in SS and other tumors contribute to tumor formation, do they do so through the same mechanism? In SS, *MNT* loss was heterozygous and the mutational status of the remaining *MNT* (or *MXD1*) allele was not interrogated. However, in studies of breast, lung and medulloblastoma tumors that often feature loss at 17p13.3, there was no clear evidence of inactivating mutations in *MNT* [44,45,46,47,48]. In medulloblastoma, heterozygous loss of *MNT* was associated with reduced MNT expression and an increase in the ratio of MYC to MNT mRNA [48]. While it would be informative to establish whether MNT expression was reduced as predicted in SS and additional tumor types with heterozygous loss at 17p13.3, the available information suggests that MNT is representative of a class of tumor suppressors defined by haplo-insufficiency and retention of an unaltered altered allele. Moreover, MXI1 and other members of the MXD family may fall in this same class of tumor suppressors.

## 5. MNT as a Facilitator of MYC-Driven Proliferation and Oncogenesis

The retention of a wildtype *MNT* allele in *MNT* heterozygous tumors raises the possibility that loss of both copies of *MNT* via deletion or inactivating mutation is not selective for oncogenesis, and may even be detrimental and selected against. Consistent with this idea, deletion of both *MNT* alleles in the MYC-driven T cell lymphoma model mentioned above caused very high levels of apoptosis and prevented tumorigenesis [49]. Thus, while loss of MNT and mild MYC overexpression act similarly to generate a balance of apoptosis and proliferation that is ultimately favorable for tumor formation, when combined, the balance is shifted steeply toward apoptosis and appears to be analogous to when MYC expression is driven above a certain threshold level to where apoptosis becomes the dominant outcome and tumor formation is abrogated [50].

Interestingly, MNT and MYC abundance appears to be regulated through a poorly-defined feedback system, with MNT being elevated by ectopic MYC overexpression [49] and MYC abundance downregulated in the absence of MNT [11,17]. In theory, MYC-dependent upregulation of MNT would provide an integrated system to buffer against the induction of excessive apoptosis in settings of high MYC and allow proliferation and cell number expansion to proceed, both normally and in the context of tumorigenesis. Evidence for such an integrated system appears to exist in the setting of T cell stimulation following engagement of the OX40 receptor [51]. OX40 activation causes a very robust proliferative response in T cells and was associated with induction and sustained expression of MYC, but also of MNT and MXD4 [51]. While the increase in MNT and MXD4 is predicted to counter the role of MYC in promoting proliferation, in the setting of T cell activation where proliferation rates are propelled to and maintained at extremely high levels, the primary function of the sustained MNT and MXD upregulation appeared to be to limit apoptosis [51]. These results suggest that upregulation of MNT (and MXD or other MYC antagonists), either directly or indirectly by MYC, may play an important cooperative role in sustaining T cell proliferation and immune responses.

**Figure 2:**
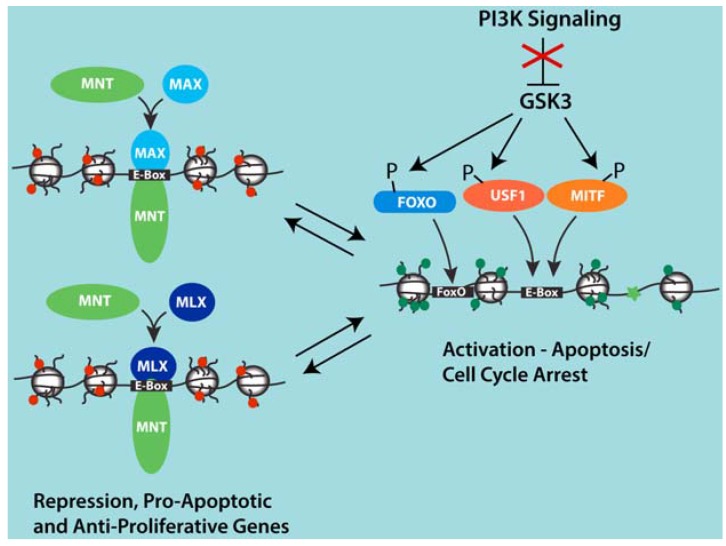
Model for competition between MNT-MAX and USF1 and MITF at a group of shared target genes involved in apoptosis and cell cycle arrest that are induced upon PI3K inhibition. Inhibition of PI3K signaling activates GSK3 and leads to the phosphorylation of USF1, MITF and FOXO transcription factors. Through mechanisms that remain unclear, phosphorylation by GSK3 is associated with the displacement of MNT-MAX with USF1 and MITF complexes at Ebox sites and the binding of FOXO factors at adjacent sites in proximal promoter regions of several genes induced by PI3K inhibition. This set of genes appears to not be regulated by MYC-MAX complexes. However, repression of these genes by MNT may cooperate with PI3K signaling and elevated MYC to promote proliferation and cell expansion by preventing apoptosis that might be otherwise sensitized due to the activities of high MYC.

The above results hint at the existence of a subset of MNT target genes whose downregulation is critical for mitigating apoptosis in populations of rapidly proliferating cells expressing high MYC. In experiments examining the transcriptional response to inhibition of pro-proliferative and survival PI3-kinase signaling, Terragni et al. showed that MNT-MAX is displaced from Ebox sites on several upregulated genes with roles in apoptosis and cell cycle regulation [52]. Interestingly, while MNT repression is associated with MNT binding to Ebox elements in these genes, they appear to not be MYC targets, but instead are targets of the BHLHZip factors Melanogenesis Associated Transcription Factor (MITF) and Upstream stimulatory factor 1 (USF1) and act in conjunction with Forkhead box (FOXO) transcription factors [52] (Figure 2). PI3-kinase/AKT signaling did not downregulate MNT or upregulate MITF, USF1 or FOXO proteins but led to the GSK3-dependent phosphorylation of the latter proteins. However, how these phosphorylation events might contribute to the switch from MNT-MAX to USF1 and/or MITF binding at Ebox sites remains unclear. Nonetheless, these findings suggest that maintaining or increasing MNT expression in highly proliferating cells may confer prosurvival activity downstream of PI3 kinase signaling through active repression of genes such as Atrogin-1 and TXNIP that can promote apoptosis [52].

It is important to note that not all pro-mitogenic conditions lead to increases in both MYC and MNT or MNT activity. In MEFs stimulated with serum to enter the cell cycle for example, MYC is induced but MNT is only weakly increased [17,19,53]. Under such conditions, the limited window of elevated MYC appears to temporarily sop up much of the available MAX and render it limiting for heterodimerization with MNT [19]. The ratio of MYC-MAX to MNT-MAX complexes (and potentially MXD/MGA-MAX complexes) appears to shift to strongly favor MYC-MAX with the effect of reducing transcriptional antagonism of shared target genes manifest as a spike in MYC-driven transcription [19]. Unlike the setting of sustained proliferation that occurs following T cell activation, in growth-factor-stimulated cell cycle entry of MEFs and potentially other cell types, the reduction in the ratio of MNT-MAX to MYC-MAX may facilitate the induction of MYC target genes that function to promote cell cycle entry while being transient enough to not trigger MYC-dependent apoptosis. Moreover, there is evidence that transient growth-factor-induced phosphorylation of MNT may interfere with the binding of SIN3 corepressors to MNT and thereby suppress its repression activity during the window of peak MYC [21]. However, persistent loss of MNT in this setting leads to apoptosis, indicating that the proper balance between MYC and MNT must be restored to progress towards sustained proliferation.

## 6. Hypoxia Regulation of MNT and MNT-MYC Antagonism

In addition to a poorly understood MYC-MNT regulatory circuit mentioned above, other regulatory mechanisms control MNT RNA and protein abundance. One intriguing mechanism is MNT downregulation by the micro RNA miR-210. mir-210 binds to a 3’ untranslated sequence of MNT mRNA and leads to reduced MNT RNA and protein [54]. miR-210 is one of the most upregulated RNAs in response to hypoxia [55] and is implicated as being a biomarker and/or causative agent in a number of diseases, including cancer and cardiovascular disease [56]. Zhang et al. showed that miR-210 is a direct target of hypoxia inducible factor HIF-1α and that miR-210-mediated downregulation of MNT is associated with the ability of miR-210 to override hypoxia-induced cell cycle arrest [54]. Ectopic miR-210 expression had the effect of substantially reversing hypoxia-induced gene expression and this effect was mimicked in part by knocking down MNT [54]. Moreover, the gene expression profiles of cells subjected to ectopic miR-210 and MYC overlapped, supporting the idea that there is substantial overlap between MNT and MYC transcriptional targets [54]. In the context of tumor formation, reduced MNT expression as a response to hypoxia is predicted to, among other things, relieve repression at target genes shared with MYC (and potentially with HIF factors) and therefore aid the expression of transcriptional programs governed MYC and HIF factors that reprogram metabolism to support glucose uptake and glycolysis utilized for macromolecule biosynthesis and proliferation in a low oxygen environment [57].

There are several studies thus far suggesting that induction of miR-210 and associated downregulation of MNT may contribute to the adaptation of tumor cells to hypoxic conditions [58,59]. For example, in a model of cholestasis-induced cholangiocarcinoma, where the toxic build-up of bile acids in the liver leads to hypoxia and hepatocellular injury as a precursor to tumorigenesis, it was found that miR-210 was induced and MNT was downregulated [20,58]. MNT downregulation was associated with a switch from MNT-MAX to MYC-MAX complexes at the *CYCLIN D1* and *TP53* promoters in hypoxic liver tissue and hepatocytes [20]. In this setting, upregulation of MYC through a Lin-28B-dependent mechanism may further contribute to the switch from MNT-MAX to MYC-MAX complexes on the CYCLIN D1 and TP53 promoters [58]. This transcriptional switch was linked to an increase in both CYCLIN D1 and TP53 and the induction of a chaotic cellular environment associated with cholestasis that features both increased apoptosis (that may be associated with increased TP53) and proliferation (that may be driven in part by increased CYCLIN D1) from which tumors emerge [20,58].

Similar to the cholestasis model, cultured glioma stem cells (GSCs) grown under hypoxic conditions induced miR-210 and downregulate MNT [59]. MNT downregulation in this setting allows continued GSC proliferation, prevents differentiation and as a result is permissive for neurosphere formation [59]. While the mechanisms by which MNT downregulation promotes these phenotypes was not demonstrated, increased MYC and HIF activity are the likely culprits. Nonetheless, the apparent targeting of MNT by hypoxia and miR-210 during cholangiocarcinogenesis and in GSCs reinforces the notion that MNT functions as a tumor suppressor.

Finally, in addition to oncogenesis, proliferation in the hypoxic setting of idiopathic pulmonary fibrosis was associated with both miR-210 induction and downregulation of MNT [60]. Thus, downregulation of MNT may be functionally important for the hypoxia response in a wide variety of injury and disease settings.

## 7. Control of MNT Degradation by E6AP

MNT was identified in a screen for proteins associated with the E3 ubiquitin ligase E6AP (E6 associated protein, also known as UBE3A) [61]. E6AP is the founding member of the HECT (homologous with E6AP C terminus) family of proteins and as its name implies, was originally identified as a protein associated with E6 proteins encoded by human papillomaviruses [62]. Several HPV strains, but most prominently HPV16 and HPV18, are oncogenic and contribute to cervical, head and neck, and potentially other cancer types [63]. The E6 protein contributes to HPV-associated oncogenesis through a number of potential mechanisms, with binding to TP53 and its E6AP-dependent ubiquitin-mediated degradation being perhaps the most important [64]. Kapoor et al. showed that E6AP also targeted MNT for ubiquitin-mediated degradation [61]. Using a myeloid differentiation assay, they found that MNT was induced upon their differentiation and that this corresponded to downregulation of E6AP and loss of E6AP-mediated degradation of MNT [61]. Like downregulation of E6AP, ectopic MNT expression also induced cell cycle arrest and myeloid differentiation. In contrast, MNT knockdown inhibited the induction of myeloid differentiation and cell cycle arrest [61]. These results suggest that E6AP expression may contribute to myeloid and potentially other cancers by maintaining low MNT levels, which in turn would reduce repression at shared MYC target genes, promote proliferation and restrict differentiation. Consistent with this idea, MYC is a potent oncoprotein in myeloid malignancies with its oncogenic activity being closely associated with the inhibition of differentiation [65]. The findings by Kapoor et al. and colleagues [61] also suggest that agents such as All-trans retinoic acid, Vitamin D3 and phorbol 12-myristate 13-acetate used for differentiation therapy in acute myeloid malignancies may act in part by inducing MNT.

## 8. Summary and Future Directions

It is becoming increasingly clear that MNT plays an integral and dose-dependent role in governing the response cells have to mitogenic signaling and MYC induction. Unlike the more tissue and context-specific expression of members of the MXD family and MGA [14,42,66,67], MNT expression is ubiquitous and repression mediated by MNT-MAX may serve to establish a ground state of expression at many MYC targets when MYC levels are low, and constrain the level of activation or transcriptional amplification upon induction of MYC. An analogous system may operate involving MNT-MLX complexes in the regulation of MLXIP-MLX target genes. Beyond the MAX and MLX interactome, MNT-MAX and MNT-MLX complexes may bind and regulate target genes not recognized by either MYC-MAX or MLXIP-MLX and instead that are positively regulated by other Ebox binding transcription factors such as USF and MITF [52]. Further interrogation of genome-wide binding by MNT and the global response of its target genes to the acute loss and gain of MNT MYC and MLXIP is needed to establish a more detailed and comprehensive picture of how MNT antagonism governs gene expression within this network. One potential outcome of such an endeavor is the identification of distinct subsets of MYC, MLXIP and other targets that are particularly sensitive to MNT repression. Such genes may define a category of genes that, when induced, play a primary and general role in initiating metabolic adaptation to hypoxia and allow the establishment and progression of incipient tumors. Moreover, given the importance of MNT in preventing apoptosis in cells with high MYC, MNT-MYC targets may be enriched in pro-apoptotic genes whose repression is required to sustain proliferation in settings such as immune responses and MYC-driven oncogenesis.
